# Asymmetric Distribution of Cadherin 23 and Protocadherin 15 in the Kinocilial Links of Avian Sensory Hair Cells

**DOI:** 10.1002/cne.22456

**Published:** 2010-07-26

**Authors:** Richard J Goodyear, Andy Forge, P Kevin Legan, Guy P Richardson

**Affiliations:** 1 School of Life Sciences, University of SussexFalmer, Brighton, BN1 9QG, United Kingdom; 2 UCL Ear InstituteUCL, 330-332 Gray's Inn Road, London, WC1 8EE, United Kingdom

**Keywords:** hair cell, mechanotransduction, stereocilia, cadherin 23, protocadherin 15

## Abstract

Cadherin 23 and protocadherin 15 are components of tip links, fine filaments that interlink the stereocilia of hair cells and are believed to gate the hair cell's mechanotransducer channels. Tip links are aligned along the hair bundle's axis of mechanosensitivity, stretching obliquely from the top of one stereocilium to the side of an adjacent, taller stereocilium. In guinea pig auditory hair cells, tip links are polarized with cadherin 23 at the upper end and protocadherin 15 at the lower end, where the transducer channel is located. Double immunogold labeling of avian hair cells was used to study the distribution of these two proteins in kinocilial links, a link type that attaches the tallest stereocilia of the hair bundle to the kinocilium. In the kinocilial links of vestibular hair bundles, cadherin 23 localizes to the stereocilium and protocadherin 15 to the kinocilium. The two cadherins are therefore asymmetrically distributed within the kinocilial links but of a polarity that is, within those links that are aligned along the hair bundle's axis of sensitivity, reversed relative to that of tip links. Conventional transmission electron microscopy of hair bundles fixed in the presence of tannic acid reveals a distinct density in the 120–130 nm long kinocilial links that is located 35–40 nm from the kinociliary membrane. The location of this density is consistent with it being the site at which interactions occur in an in trans configuration between the opposing N-termini of homodimeric forms of cadherin 23 and protocadherin 15. J. Comp. Neurol. 518:4288–4297, 2010. © 2010 Wiley-Liss, Inc.

The mechanosensory hair bundles of the hair cells in the inner ear and the lateral line organs of vertebrates are comprised of three or more height-ranked rows of stereocilia and, in most cases, a single kinocilium. Up to four morphologically and biochemically distinct types of link (tip links, horizontal top connectors, shaft connectors, and ankle links) interconnect adjacent stereocilia, and the kinocilium is coupled to the adjacent stereocilia by numerous fine strands known as kinocilial links.

Tip links are, in mature animals, uniquely aligned along the hair bundle's axis of mechanosensitivity, stretching from the top of a stereocilium to the side of an adjacent, taller stereocilium (Pickles et al., 1984). These tip links are widely believed to gate, either directly or indirectly, the hair cell's as-yet unidentified mechanotransducer channel, a calcium selective cation channel (Lumpkin et al., 1997) that is now known to be located at the lower end of the tip link in mammalian cochlear hair cells (Beurg et al., 2009). The other links, often collectively referred to as lateral links, are not restricted in their orientation with respect to the hair bundle's axis of sensitivity and are generally thought to hold the individual elements of the hair bundle together so that it operates as a coherent unit. These links may also play major roles in the development and maintenance of hair-bundle structure (Frolenkov et al., 2004; Nayak et al., 2007, Petit and Richardson, 2009).

Many of the proteins associated with these different link types have now been identified, either by the use of monoclonal antibodies derived from mice immunized with membrane fractions derived from the inner ear (Richardson et al., 1990; Goodyear and Richardson, 1992, 1999, 2003; Goodyear et al., 2003; McGee et al., 2006; Ahmed et al., 2006) or as the products of various deafness loci (Söllner et al., 2004; Siemens et al., 2004; Adato et al., 2005; Michalski et al., 2007; Kazermierczak et al., 2007; Verpy et al., 2008). Cadherin 23 and protocadherin 15 are components of the tip links (Söllner et al., 2004; Siemens et al., 2004; Ahmed et al., 2006; Kazermierczak et al., 2007), the very large G-protein coupled receptor (Vlgr1) and usherin are associated with the ankle links (Adato et al., 2005; McGee et al., 2006; Michalski et al., 2007), Ptprq is a component of the shaft connectors (Goodyear et al., 2003), and stereocilin is, in mammalian cochlear outer hair cells, located in the region of the top connectors (Verpy et al., 2008).

Kinocilial links were described in some of the earliest ultrastructural studies of the hair bundle (Hillman, 1969; Hillman and Lewis, 1971). In frogs they form a meshwork connecting the kinocilial bulb to the upper ends of the immediately adjacent stereocilia. In species in which there is not a bulb at the end of the kinocilium, the kinocilial links connect a long region of the axonemal membrane to the nearest stereocilia. The similarity between tip and kinocilial links was first noted when it was shown that the two structures shared a common antigen (the tip-link antigen [TLA], now known to be protocadherin 15) and were found to have similar properties, with both links being subtilisin-resistant and sensitive to calcium chelation (Goodyear and Richardson, 2003). Cadherin 23 also localizes to the region of the kinocilial links (Siemens et al., 2004; Lagziel et al., 2005), and Fourier analysis of transmission electron micrographs revealed kinocilial links have a similar structure to that of tip links (Tsuprun et al., 2004).

In guinea pig outer hair cells, cadherin 23 localizes to the upper end of the tip link and protocadherin 15 to the lower end (Kazermierczak et al., 2007). The polarity of the two cadherins within the tip link indicates that protocadherin 15, rather than cadherin 23, may interact directly with the hair cell's mechanotransducer channel. Mature cochlear hair cells do not possess a kinocilium, and the relative distribution of the cadherins within the kinocilial links of sensory hair cells remains to be addressed. Such information may help inform as to the function of kinocilial links and their possible roles in mechanotransduction. Double immunolabeling techniques were therefore used to examine and compare the distribution of protocadherin 15 and cadherin 23 in the tip and kinocilial links of hair cells in the avian inner ear. The results indicate that the polarity of the two cadherins within the kinocilial links that are aligned along the hair bundle's axis of mechanosensitivity is the reverse of that seen in tip links.

## MATERIALS AND METHODS

### Animals

One-day-old chicks were obtained from Joice and Hill Poultry (Peterborough, UK) and housed in accordance with UK Home Office regulations and with the approval of the local animal care and use committee. Animals were killed by exposure to a rising concentration of CO_2_ according to UK Home Office guidelines.

### Antibodies and their characterization

Antibodies used are listed in Table 1. Monoclonal antibody (mAb) G19 is an IgG_1_ class antibody that recognizes an epitope located in the ectodomain of avian protocadherin 15 (Goodyear and Richardson, 2003; Ahmed et al., 2006). Mab G19 specifically immunoprecipitates bands of 200 and 250 kDa from detergent lysates of chicken inner ear sensory organs (Goodyear and Richardson, 2003), both of which are identified as protocadherin 15 by proteomic analysis (Ahmed et al., 2006). R805 is a rabbit polyclonal antibody raised to a recombinant fragment of avian cadherin 23 encompassing the 5th and 6th cadherin repeats. Briefly, primers Ggcadherin23F4 (GCAGCCATATGCTCTTTGCGAATGAGAGCAT, NdeI site underlined) and Ggcadherin23R4 (CAGCCGGATCCTCAGTAG TTGTCATTGATGTCCA, BamHI site underlined) were used to amplify a 453-basepair (bp) region of chicken cadherin 23 from ChEST clone 597C19 using Pfu polymerase (Stratagene, Netherlands). The product spans amino acids 437–781 of the predicted chicken cdh23 (XP_421595) and was cloned into the NdeI and BamHI sites of pET15b to produce a protein fused at its N-terminus with a poly-histidine sequence. The 6His-tagged fusion protein was expressed in *E. coli* BL21(DE3)pLysS and purified by Ni^2+^ affinity chromatography. Rabbit antisera were generated commercially (Eurogentec, Belgium) and affinity-purified on recombinant fusion protein coupled to CNBr activated Sepharose 4B. Antibody Ela3N to mouse cadherin 23 was a kind gift from Dr. Aziz El-Amraoui and Prof. Christine Petit (Institut Pasteur, Paris, France). To verify and confirm the specificity of the affinity-purified rabbit antibodies to cadherin 23, inner ears from early postnatal (P0–P2) waltzer v2J mouse pups were fixed for 1 hour in 4% paraformaldehyde in 0.1 M sodium phosphate pH 7.4 and washed three times in phosphate-buffered saline (PBS). Cochlear coils were dissected, preblocked in tris-buffered saline [TBS] with 10% heat-inactivated horse serum (TBS/HS), and stained overnight with affinity-purified R805 or Ela3N (Michel et al., 2005) in preblock containing 2 mM EDTA. Following washing to remove unbound antibodies, tissues were labeled with Alexa-488 conjugated goat antirabbit and Texas Red conjugated phalloidin for 2 hours, washed, mounted in Vectashield and observed with a Zeiss LSM510 confocal microscope using a 100× oil immersion lens NA 1.4. Tissues from the waltzer v2J pups were kindly provided and genotyped by Jennifer Hilton and Prof. Karen Steel (Wellcome Trust Sanger Institute, Cambridge, UK).

**1:** Primary Antibodies Used

Antigen	Immunogen	Manufacturer	Dilution used
Protocadherin 15	Chick inner ear membranes	Mouse monoclonal antibody (G19) produced by Goodyear and Richardson (2003)	1:10 (tissue culture supernatant)
Cadherin 23 (chicken)	Recombinant fragment (aa 437-781) of the predicted chicken cadherin 23 sequence (XP_421595) fused to a poly-histidine tag	Polyclonal antibodies raised in rabbit by Eurogentec, Belgium (R805)	1:50-1:100
Cadherin 23 (mouse)	Peptide sequences derived from ectodomain of mouse cadherin 23 (NH2-CRGPRPLDRERNSSH-COOH and NH2-GDISVLSSLDREKKDH-COOH derived from exons 29 and 52 respectively) conjugated to keyhole limpet hemocyanin	Polyclonal antibodies (Ela3N) raised in rabbit by Covalab, Lyon, France. (Michel et al., 2005)	1:100

### Immunolabeling

Avian inner ear tissues were obtained from 2–7-day-old chicks. Inner ears were dissected in PBS pH 7.2, fixed in 4% paraformaldehyde buffered with 0.1 M sodium phosphate buffer pH 7.4 for 1–2 hours at room temperature, and washed in PBS. Otoconial membranes with adherent otoconia were removed from utricular maculae with fine forceps prior to fixation; tectorial membranes were removed from the basilar papillae after fixation. Washed tissue pieces were incubated in preblock (TBS/HS) for 1 hour and then in preblock containing 2 mM EDTA and a mixture of mAb G19 and R805 overnight. After washing in TBS, tissues were labeled with either fluorescent or gold conjugated secondary antibodies. In some experiments staining was performed in the absence of EDTA.

For confocal microscopy, tissues were labeled with a mixture of Alexa-Fluor 488 goat antimouse and Alexa-Fluor 555 donkey antirabbit IgG, both at a dilution of 1:500 in preblock containing 0.1% TX-100 and Alexa-Fluor 350 phalloidin.

For immunogold transmission electron microscopy, tissues were labeled with a mixture of 5 nm gold antirabbit IgG and 10 nm gold antimouse IgG. For immunogold scanning electron microscopy tissues were labeled with a mixture of 20 nm gold antirabbit IgG and 10 nm gold antimouse IgG.

Fluorescently labeled tissues were mounted in Vectashield and viewed with a Zeiss LSM510 confocal microscope using a 100× Planapochromat objective, NA 1.4.

For transmission electron microscopy, gold-labeled tissues were washed, refixed in 2.5% glutaraldehyde in 0.1 M cacodylate buffer pH 7.2 containing 1% tannic acid, washed in buffer, and postfixed in 1% osmium tetroxide. After a brief wash with H_2_O, samples were dehydrated through increasing concentrations of ethanol and imbedded in TAAB 812 resin. Thin sections were cut with a diamond knife, mounted on copper mesh grids, double stained with uranyl acetate and lead citrate, and viewed in a Hitachi 7100 electron microscope operating at 100 kV. Images were captured with a Gatan camera at 2048 × 2048 pixel resolution.

For scanning electron microscopy, gold-labeled tissues were washed, refixed in 2.5% glutaraldehyde, osmicated, dehydrated with ethanol, and critical point-dried from liquid CO_2_. After rotary evaporative carbon coating, the tissue samples were examined in a field emission Jeol 6700F SEM using secondary and backscatter electron detectors. For conventional transmission electron microscopy, tissues were prepared as described previously (Goodyear and Richardson, 1992).

Figures were constructed using Adobe Photoshop CS4 (San Jose, CA) and minor adjustments to image contrast and brightness were made to some images.

## RESULTS

### Properties of the protocadherin 15 and cadherin 23 antibodies

**Figure 1:**
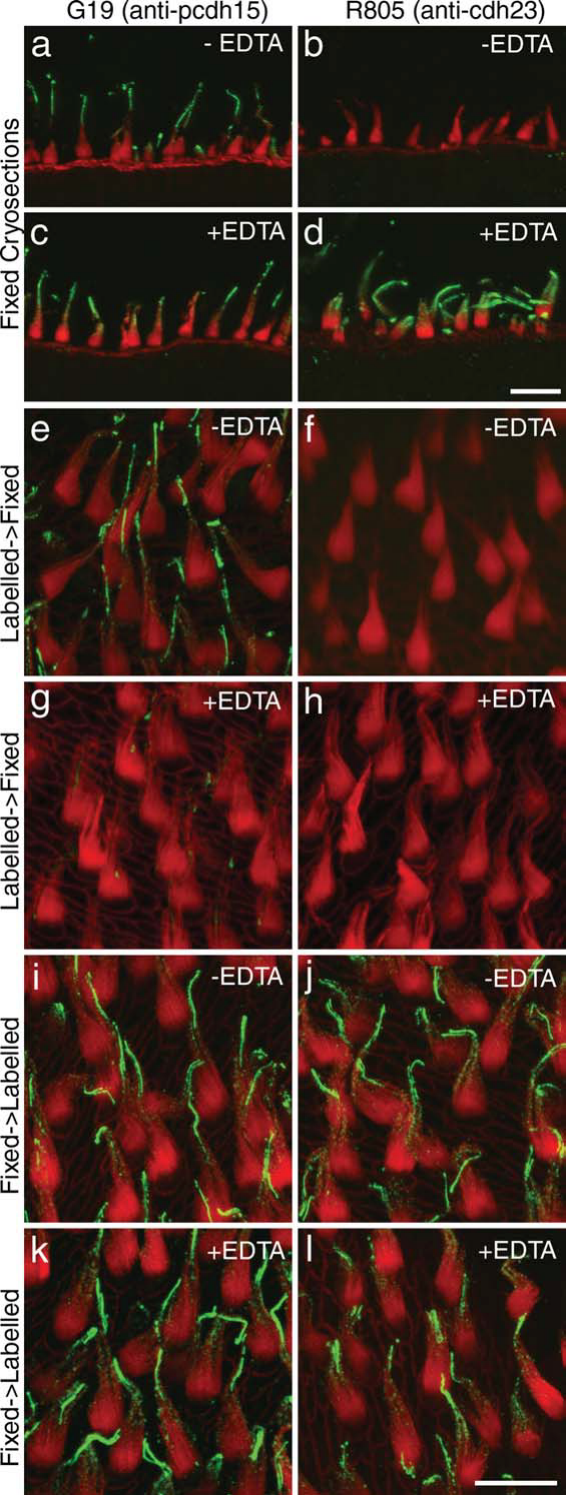
Confocal images of cryosections (**a–d**) and utricular wholemounts (**e–l**) double-labeled with mAb G19 recognizing protocadherin 15 (green in a,c,e,g,i,k) or R805 recognizing cadherin 23 (green in b,d,f,h,j,l) and Texas-red conjugated phalloidin (red). Maculae in e–h were labeled with primary antibody prior to fixation, maculae i–l were labeled after fixation. Sections and maculae were labeled in the presence (c,d,g,h,k,l) or absence (a,b,e,f,i,j) of 2 mM EDTA. A magenta-green version of this figure is available as Supporting Figure 1. Scale bars = 10 μm.

Many antibodies to cadherin 23 and protocadherin 15, especially those raised to peptides or recombinant fragments, only stain hair bundles if the epitopes are unmasked. Figure 1 compares how the two antibodies used in this study behave when used to label cryosections of formaldehyde-fixed tissue (Fig. 1a–d), live maculae prior to fixation (Fig. 1e–h), or formaldehyde-fixed macular wholemounts (Fig. 1i–l) in either the presence or absence of the divalent cation chelator EDTA. mAb G19 stains hair bundles in all three types of preparation (Fig. 1a,c,e,g,i,k), irrespective of the presence or absence of EDTA, although the extent of labeling is considerably reduced when maculae are labeled in the presence of EDTA before fixation (Fig. 1g). This is expected, as mAb G19 has been previously shown to recognize a Ca^++^-dependent epitope (Goodyear and Richardson, 2003). In contrast, R805 stains hair bundles in formaldehyde-fixed cryosections only in the presence (Fig. 1d) and not in the absence (Fig. 1b) of EDTA, and it does not stain hair bundles in live maculae in either the presence (Fig. 1h) or absence (Fig. 1f) of EDTA. Following fixation, hair bundles in macular wholemounts stain irrespective of the presence or absence of EDTA (Fig. 1j,l). These observations indicate that the cadherin 23 epitopes recognized by R805 are 1) masked in cryosections of fixed tissues and in live tissues, 2) can be unmasked by EDTA in cryosections but not in live macular wholemounts, and (3) can be unmasked by fixation in wholemounts. Some aspect of the cryosection preparation procedure, possibly drying the frozen sections onto the glass slides, appears to mask epitopes after fixation and EDTA is then required to reveal them. These observations reveal the complexities that can be encountered when using antibodies directed against these cell-surface antigens of the hair bundle and indicate negative results should always be treated with caution.

**Figure 2:**
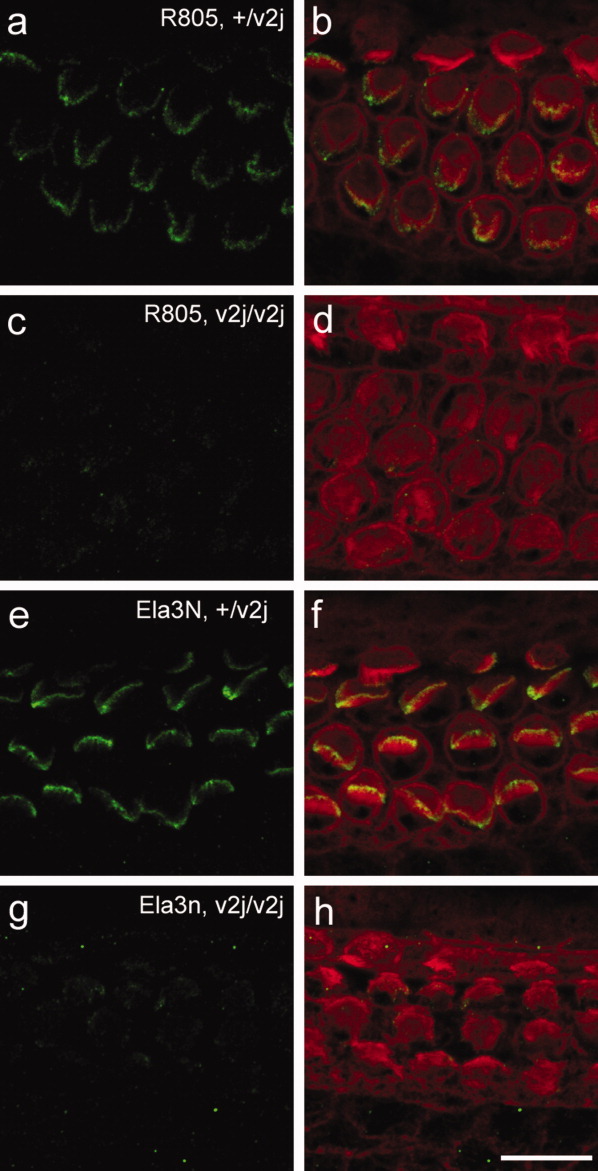
Confocal images of mouse cochlear wholemounts from +/v2J (**a,b,e,f**) and v2J/v2J (**c,d,g,h**) P2 mouse pups double-labeled with R805 raised to chick cadherin 23 (green in a–d) or Ela3N raised to mouse cadherin 23 (green in e–h) and Texas red phalloidin (red in b,d,f,h). A magenta-green version of this figure is available as Supporting Figure 2. Scale bar = 10 μm.

Specificity of the R805 antibodies was tested by staining cochlear tissues from early postnatal waltzer v2J mice. The waltzer v2J mouse has a single base mutation at the first nucleotide of intron 32 that alters a splice donor site. Most transcripts produced from this allele introduce premature stop codons (DiPalma et al., 2001), and a previous study has shown that a number of cadherin 23 antibodies show little if any staining in cochlear cultures prepared from homozygous v2J/v2J mice (Michel et al., 2005). With both R805 (Fig. 2a–d) and the previously validated antibody to a mixture of peptides in the extracellular region of the mouse cadherin 23 sequence, Ela3N (Fig. 2e–h), hair-bundle staining in the homozygous v2J/v2J mutants (Fig. 2c,d,g,h) was reduced to very low levels in comparison with that observed in the heterozygotes (Fig. 2a,b,e,f). With both antibodies some feint, low-level residual staining was observed that may be cytoplasmic.

### Discrete cadherin 23 and protocadherin 15 spots can be resolved within hair bundles by confocal microscopy

**Figure 3:**
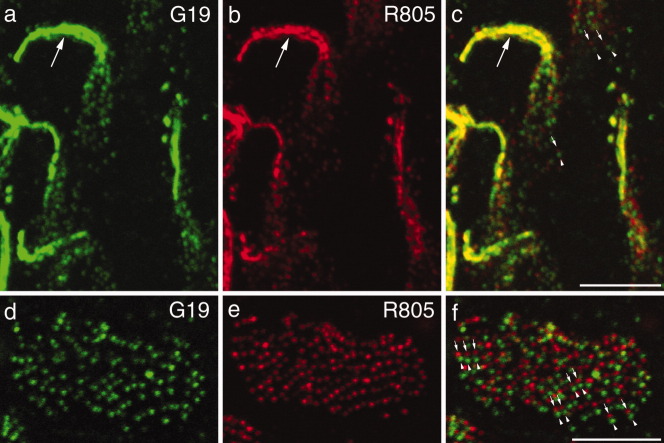
Confocal images of hair bundles from the utricular macula (**a–c**) and the basilar papilla (**d–f**) double-labeled with mAb G19 (a,d) and R805 (b,e). Corresponding merges are shown in panels c,f. A magenta-green version of this figure is available as Supporting Figure 3. Scale bars = 5 μm.

Confocal fluorescent images of avian vestibular and auditory hair bundles that have been double-labeled with mAb G19 and R805 are shown in Figure 3. In the kinocilial link region at the upper end of the vestibular hair bundles, protocadherin 15 and cadherin 23 have overlapping distributions (Fig. 3a–c). Lower down over the bevelled edge of the hair bundle discrete spots of protocadherin 15 and cadherin 23 are visible that can be resolved from one another and show little overlap in the merged images (Fig. 3a–c). In the hair bundles of the basilar papilla, discrete spots of cadherin 23 and protocadherin 15 are also visible over the inclined surface of the hair bundle (Fig. 3d–f). Similar numbers of spots are seen for each protein and a proportion of these spots lie in close proximity to one another.

### Double immunogold labeling for cadherin 23 and protocadherin 15 in tip and kinocilial links

**Figure 4:**
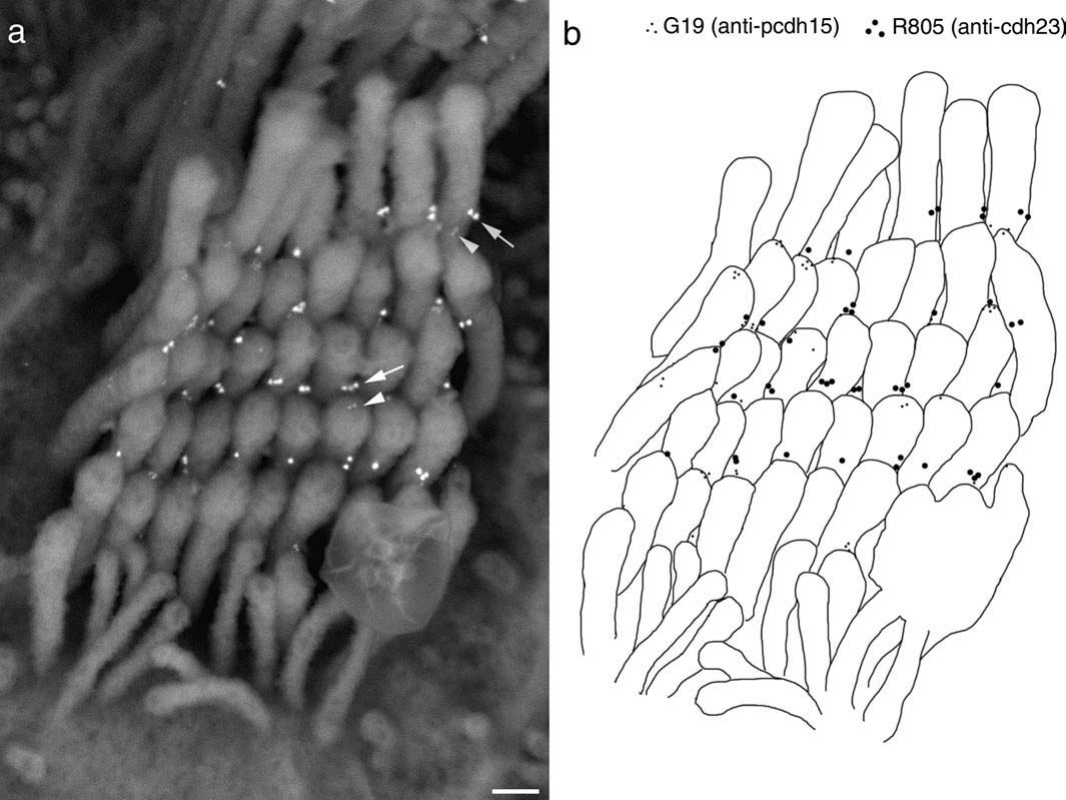
Scanning electron micrograph (**a**) taken in the backscatter mode of detection of a utricular hair bundle double labeled with mAb G19 (10 nm gold particles, arrowheads) and R805 (20 nm gold particles, arrows). A drawing of the same image is shown in panel **b** in which the 20 nm gold particles (cadherin 23) are depicted as large black dots and the 10 nm gold particles (protocadherin 15) are shown as small dots. Scale bar = 200 nm.

Scanning electron microscopy of double immunogold-labeled utricular hair bundles reveals protocadherin 15 and cadherin 23 localize close to the tips of the stereocilia (Fig. 4a). In instances where both proteins can be detected at the same potential tip-link site, protocadherin 15 lies closer to the tip of the shorter stereocilium and cadherin 23 lies closer to the side of the adjacent taller one (Fig. 4b).

**Figure 5:**
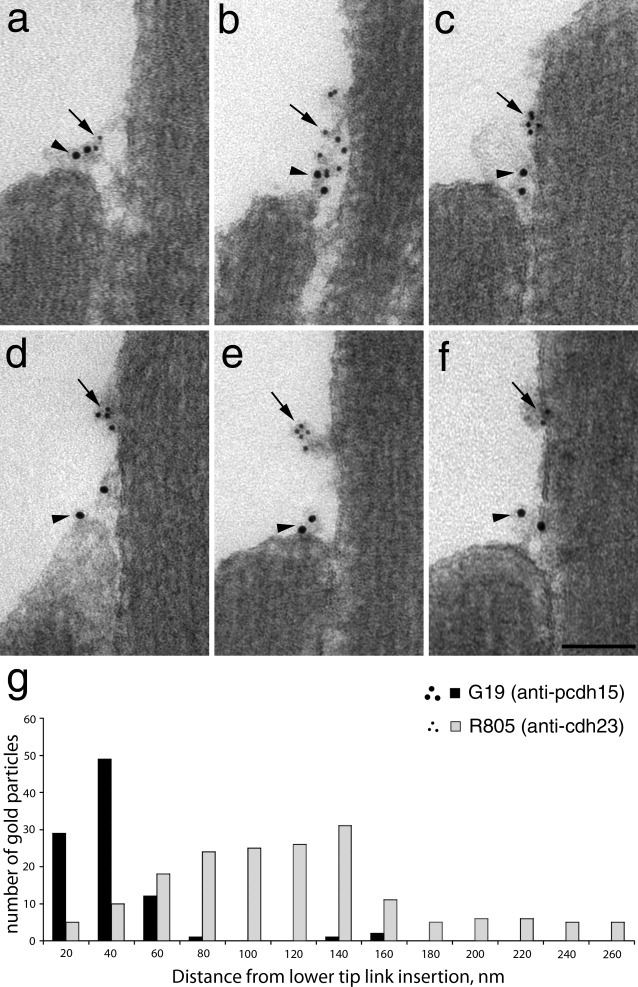
**a–f**: Transmission electron micrographs of the tip link region of utricular hair bundles double immunogold-labeled with mAb G19 (10 nm gold particles, arrowheads) and R805 (5 nm gold particles, arrows). The images are representative of the spread of gold particles seen in the tip link region with panel a showing the closest apposition of the two labels and panel f showing the greatest separation. Scale bar = 100 nm. **g**: Histogram showing the distances at which gold particles labeling protocadherin 15 (black bars) and cadherin 23 (gray bars) on macular hair cells are located from the lower insertion point of the tip link. Scale bar = 100 nm.

Transmission electron micrographs of the tip-link region from vestibular hair bundles that have been double immunogold-labeled with mAb G19 and R805 are shown in Figure 5a–f. These images are representative of the spread of labeling observed. A quantitative analysis of the distances at which the two different-sized gold particles are located relative to the top of the shorter stereocilium in these tip-link regions (Fig. 5g) indicates that the protocadherin 15 epitope recognized by mAb G19 localizes closer to the tip of the short stereocilium, while the cadherin 23 epitopes seen by R805 lie more distant. On average, the gold particles labeling protocadherin 15 are located 31.4 ± 24.2 nm (n = 94) from the tip of the shorter stereocilium and the gold particles labeling cadherin 23 are found at a distance of 110.4 ± 54.8 nm (n = 177).

**Figure 6:**
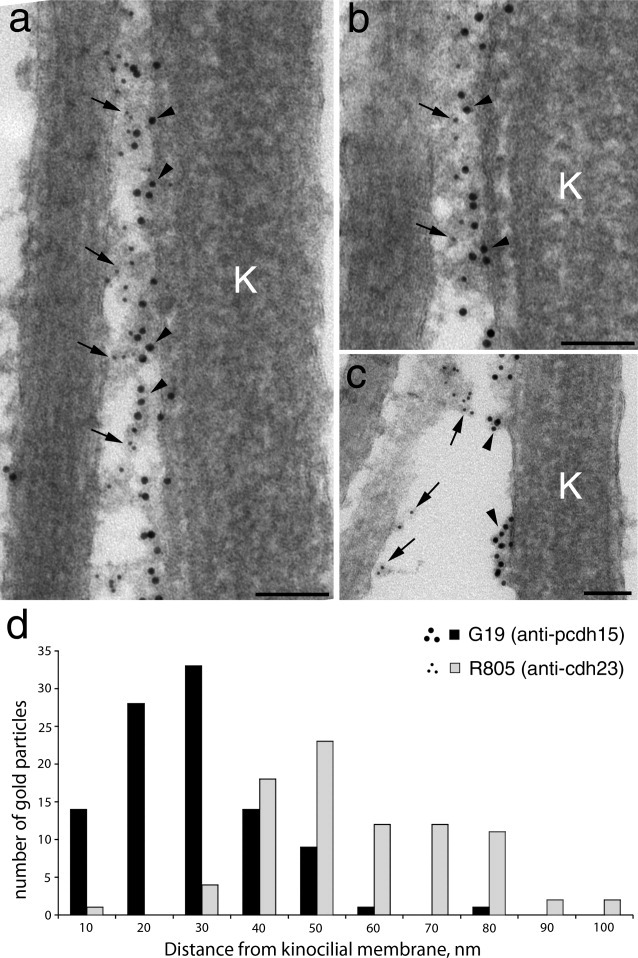
**a–c**: Transmission electron micrographs of the kinocilial link region of utricular hair bundles double immunogold-labeled with mAb G19 (10 nm gold particles, arrowheads) and R805 (5 nm gold particles, arrows). In panel c the kinocilium has separated from the stereocilia. K = kinocilium. **d**: Histogram showing the distances at which gold particles labeling protocadherin 15 (black bars) and cadherin 23 (grey bars) on macular hair cells are located from the kinocilial membrane. Scale bars = 100 nm.

In the kinocilial link region, double immunogold labeling indicates that cadherin 23 lies closer to the stereocilium, while protocadherin 15 is nearer to the kinocilium (Fig. 6a,b). In instances in which the kinocilium is fortuitously separated from the stereocilia, label for protocadherin 15 is associated with the kinocilium, whereas label for cadherin 23 is associated with the nearby stereocilia (Fig. 6c). A quantitative analysis of gold particle distribution in the kinocilial link region (Fig. 6d) reveals cadherin 23 lies further away from the kinocilium, whereas protocadherin 15 lies nearer to it. On average, the gold particles labeling protocadherin 15 are located 23.4 ± 12.6 nm (n = 100) from the membrane of the kinocilium and the gold particles recognizing cadherin 23 are found at a distance of 51.5 ± 17.0 nm (n = 85).

### Fine structure of kinocilial links reveals periodic striations

**Figure 7:**
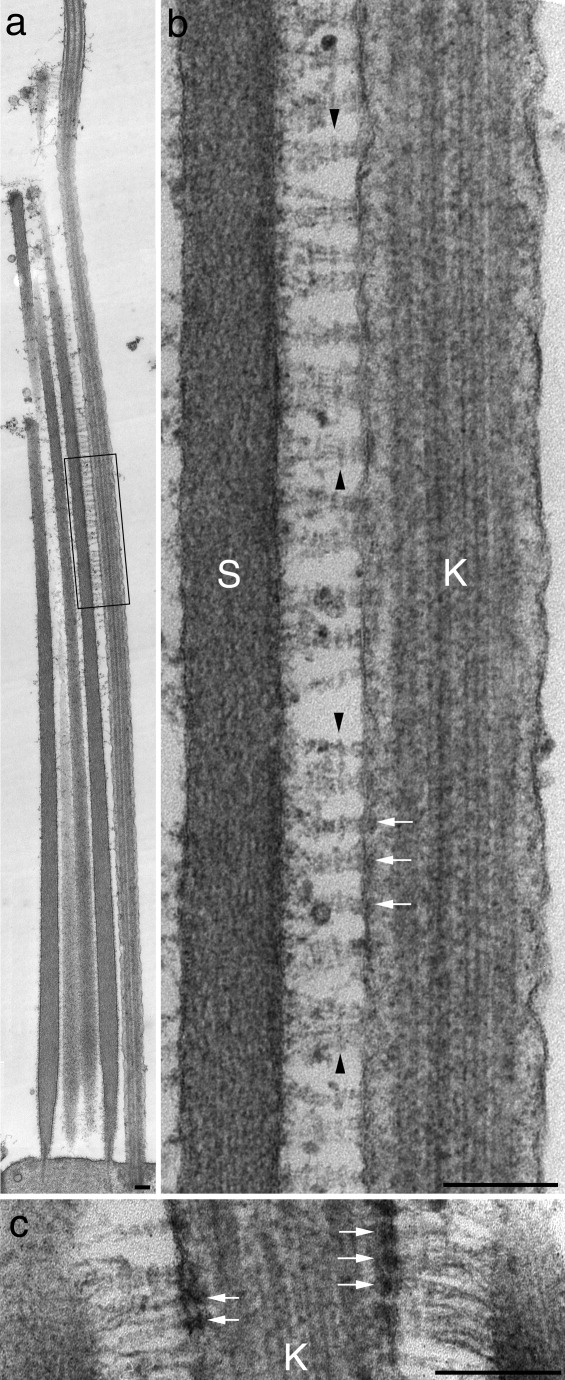
**a,b**: Transmission electron micrographs of a hair bundle from a utricular macula that was fixed in the presence of tannic acid. The region boxed in panel a is shown at higher magnification in panel b. Note the prominent density (arrowheads) along the kinocilial links that is situated 35–40 nm from the kinocilial membrane. Submembranous densities at link attachment sites in the kinocilium are indicated by arrows. **c**: Detail from a kinocilial–stereocilial junction showing the prominent submembranous densities (arrows) within the kinocilium at the attachment sites of the kinocilial links. K = kinocilium, S = stereocilium. Scale bars = 200 nm.

Conventional transmission electron micrographs of preparations that have been fixed and prepared for electron microscopy in the presence of the mordant tannic acid, an agent that enhances the binding of osmium to structures and therefore their electron density (Simionescue and Simionescue, 1976a, b), reveal the presence of periodic striations along the length of the kinocilial links (Fig. 7a,b). A particularly dense and prominent striation is observed at a distance of 35–40 nm from the membrane of the kinocilium and no further striations are consistently observed between this point and the membrane of the kinocilium. Submembranous densities are also observed within the kinocilium at the sites at which the kinocilial links are attached (Fig. 7b,c).

## DISCUSSION

Although previous reports have shown cadherin 23 (Siemens et al., 2004; Lagziel et al., 2005; Shin et al., 2005) and protocadherin 15 (Goodyear and Richardson, 2003) are both associated with the kinocilial links, the polarity of the two proteins within these links has remained hitherto unresolved. Immunofluorescence studies of immature cochlear hair bundles (Lagziel et al., 2005; Michel et al., 2005) and relatively mature ampullary bundles in mammals (Lagziel et al., 2005) have suggested that cadherin 23 is expressed by kinocilia. In contrast, immunofluorescence microscopy of (presumably mature) frog saccular hair cells indicates cadherin 23 may only be present on the stereocilia that lie adjacent to the kinocilium (Siemens et al., 2004; Shin et al., 2005). Our current immunogold labeling in the mature hair cells of the avian inner ear reveals that protocadherin 15 and cadherin 23 are asymmetrically distributed in the kinocilial links of avian vestibular hair bundles. Protocadherin 15 is associated with the membrane of the kinocilium, while cadherin 23 is associated with the membranes of the adjacent stereocilia. Our finding that cadherin 23 is not expressed on the surface of kinocilia in mature avian utricular hair bundles is therefore in accord with the results described by Shin et al. (2005) for frog saccular hair cells.

The results also show that in chick hair cells, as in outer hair cells of the guinea pig cochlea (Kazermierczak et al., 2007), protocadherin 15 lies at the lower end of the tip link, whereas cadherin 23 lies at the upper end. Although kinocilial links do not lie strictly along the hair bundle's preferred axis of mechanosensitivity, the polarity of cadherin 23 and protocadherin 15 in the kinocilial links that are aligned along this axis is the reverse of that seen in the tip links, with the N-termini of the molecules pointing in opposite directions within the two types of links. The mechanisms that determine the polarity of tip and kinocilial links remain unknown. Multiple splice variants of protocadherin 15 are expressed in the inner ear, and the different isoform classes encoded by these variants are targeted to different compartments of the hair bundle (Ahmed et al., 2006). To generate kinocilial links of the observed asymmetry and polarity one can speculate that a protocadherin 15 isoform class specific for this type of link is excluded from the stereocilia of the hair bundle, while cadherin 23 is restricted from entering the kinocilium.

As the hair cell's mechanotransducer channel has now been shown to lie at the lower end of the tip link (Beurg et al., 2009), and as this part of the tip link is formed by protocadherin 15 (Kazermierczak et al., 2007), it is possible that this channel is directly associated with protocadherin 15. The finding reported herein that protocadherin 15 localizes to the axonemal side of the kinocilial link raises the intriguing possibility that a transducer channel may also be located in the kinocilium. Indeed, the hair-cell mechanotransducer channel candidate TRPN, originally identified in zebrafish (Sidi et a., 2003), has been found to localize to the kinociliary bulb of frog saccular hair cells (Shin et al., 2005). Furthermore, conspicuous submembranous densities, potentially analogous to the lower tip link density seen at tips of stereocilia, are found at the points where kinocilial links attach to the kinocilary bulb in frogs (Hillman and Lewis, 1971) and to the kinocilium in chicks (see Fig. 7c; Goodyear and Richardson, 2003).

The following points, however, need to be considered. First, there is no evidence that the kinocilium is required for mechanotransduction in hair cells. A kinocilium is not present in mature mammalian hair cells and the deliberate removal or dissociation of the kinocilium from frog saccular hair bundles (Hudspeth and Jacob, 1979) does not prevent transduction by the hair bundle. Second, TRPN has not been found yet in the genome of mammals and birds. Third, many of the proteins that are associated with the lower tip-link density, such as whirlin, myo15a, and espin1 (Belyantseva et al., 2003; Delprat et al., 2005; Salles et al., 2009) have not yet been reported to be present in either the kinocilium or kinociliary bulb. Fourth, at least in mammals, a member of the protocadherin 15 isoform class CD2 is associated with the kinocilium of mature mammalian vestibular hair cells, whereas a member of the protocadherin isoform class CD3 localizes to the tips of stereocilia (Ahmed et al., 2006), i.e., to sites that include those at which the channel has now been shown to be located (Beurg et al., 2009). It is not yet known which protocadherin 15 isoform class is present in the kinocilium of chick vestibular hair cells. mAb G19 recognizes an ectodomain epitope that is common to at least the CD1 and CD3 isoforms, and may also be present in the hybrid CD2/3 isoform class of protocadherin 15 that is expressed in the chick (Ahmed et al., 2006). The latter hybrid isoform, rather than the tip-link associated CD3 isoform, may be present on the axonemal side of the kinocilial link and there is therefore no a priori reason why this should be directly associated with the tip-link mechanotransducer channel. The points listed above therefore raise some reservations about the potential functionality of kinocilial links as part of a parallel mechanotransduction system and it may well be that their primary role is to transmit forces applied to the tip or bulb of the kinocilium to the stereocilia.

The current model for tip link structure suggests it is formed from cis-homodimers of protocadherin 15 and cadherin 23 that interact in trans via the N-termini of the four cadherins. The two cadherins each form parallel homodimers in vitro (Kazmierczak et al., 2007) and their lengths are similar to those predicted from the number of cadherin repeats predicted in their ectodomains (27 for cadherin 23 and 11 for protocadherin 15) and the known size of such a repeat (4 nm). In such a model the maximum length of a tip link is ≍170 nm. In light of their similar molecular composition, the structure of kinocilial links is likely to be the same as that of tip links. Unlike tip links, however, kinocilial links have a distinct striated appearance in preparations that have been fixed in tannic acid. These striations may be due to the large numbers of parallel filaments that are present in the kinocilial link region and the projection averaging that results from section thickness and the large depth of field provided by the electron microscope. In kinocilial links that were 120–130 nm long the most prominent density is located 35–40 nm from the axonemal membrane, with no other striations being typically observed between this point and the kinocilium. The position of this density, at a site ≍30% of the total link length from the axonemal membrane would be consistent with it representing the point at which the N-termini of the protocadherin 15 and cadherin 23 homodimers interact. The additional striations observed between this point and the membrane of the stereocilium might likewise represent sites at which the cadherin 23 ectodomains are interacting in cis to stabilize the links.

## Supporting information

Supplemental Figure 1. (Corresponding to Text Figure 1) Confocal images of cryosections (a-d) and utricular wholemounts (e-l) double-labelled with mAb G19 recognising protocadherin 15 (green in a, c, e, g, i, k) or R805 recognising cadherin 23 (green in b, d, f, h, j, l) and Texas-red conjugated phalloidin (magenta). Maculae in e-h were labelled with primary antibody prior to fixation, maculae i-l were labelled after fixation. Sections and maculae were labelled in the presence (c, d, g, h, k, l) or absence (a, b, e, f, i, j) of 2 mM EDTA. Bars = 10 μm.

Supplemental Figure 2. (Corresponding to Text Figure 2) Confocal images of mouse cochlear wholemounts from +/v2J (a, b, e, f) and v2J/v2J (c, d, g, h) P2 mouse pups double labelled with R805 raised to chick cadherin 23 (green in a-d) or Ela3N raised to mouse cadherin 23 (green in e-h) and Texas red phalloidin (magenta in b, d, f, h). Bar = 10 μm.

Supplemental Figure 3. (Corresponding to Text Figure 3) Confocal images of hair bundles from the utricular macula (a-c) and the basilar papilla (d-f) double labelled with mAb G19 (green in a, d) and R805 (magenta in b, e). Corresponding merges are shown in panels c & f. Bars = 5 μm.
