# Supplementary figures and images for: Asymmetric Distribution of Cadherin 23 and Protocadherin 15 in the Kinocilial Links of Avian Sensory Hair Cells
Source: J Comp Neurol. 2010 Jul 26;518(21):4288–97. doi: 10.1002/cne.22456 (PMC3337639; doi:10.1002/cne.22456)

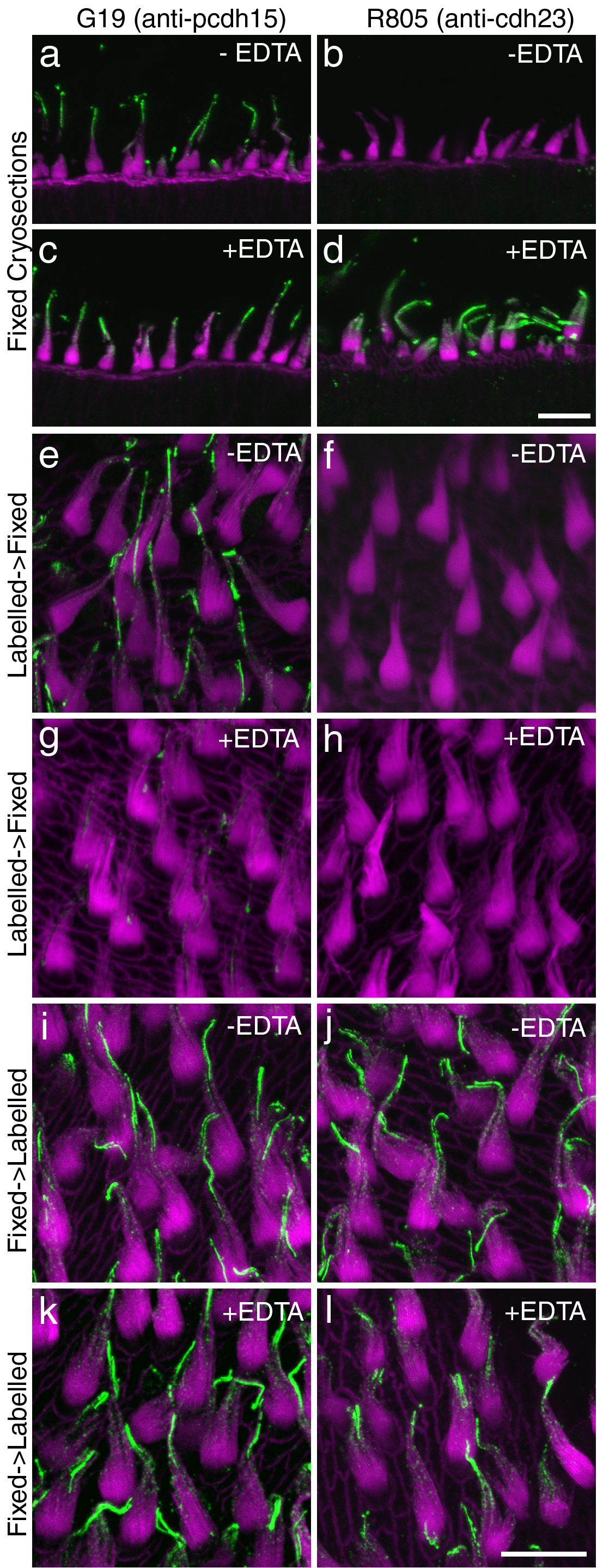

Supplement: Supplementary file 1 [file cne0518-4288-SD1.tif]

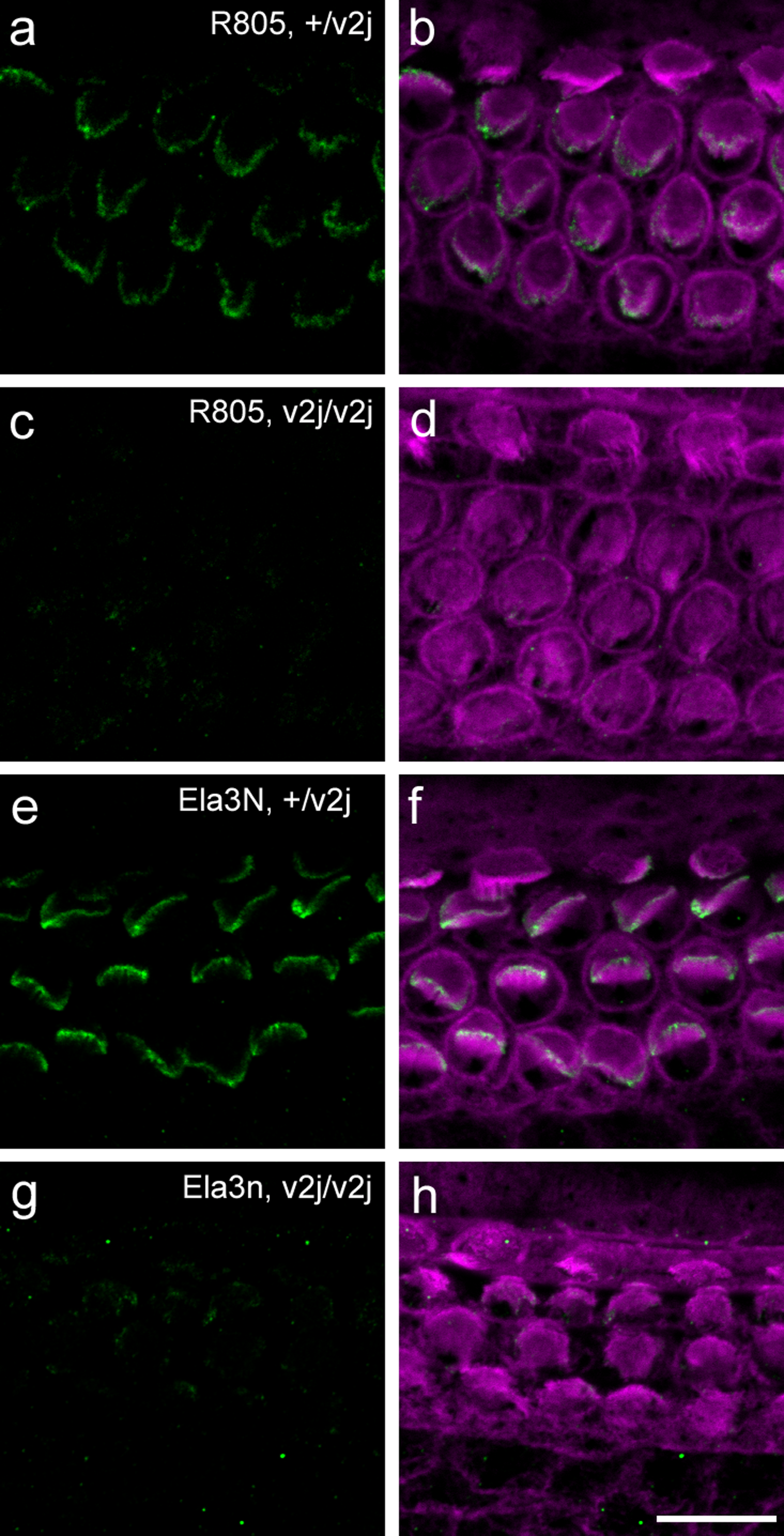

Supplement: Supplementary file 2 [file cne0518-4288-SD2.tif]

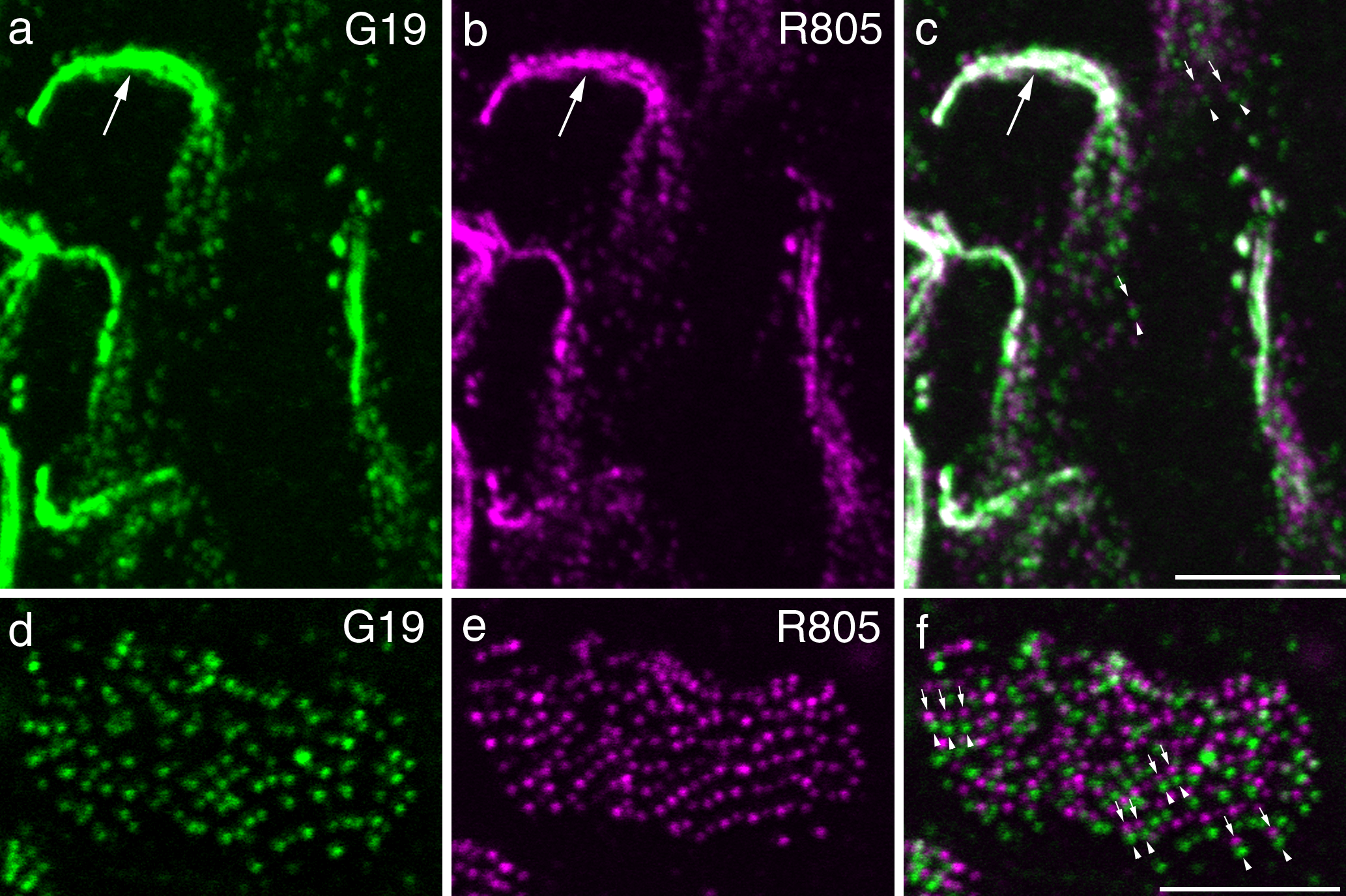

Supplement: Supplementary file 3 [file cne0518-4288-SD3.tif]
